# Evaluation of effect of low-level laser therapy on adolescents with temporomandibular disorder: study protocol for a randomized controlled trial

**DOI:** 10.1186/1745-6215-14-229

**Published:** 2013-07-22

**Authors:** Camila Haddad Leal de Godoy, Paula Fernanda da Costa Silva, Deise Sales de Araujo, Lara Jansiski Motta, Daniela Aparecida Biasotto-Gonzalez, Fabiano Politti, Raquel Agnelli Mesquita-Ferrari, Kristianne Porta Santos Fernandes, Regiane Albertini, Sandra Kalil Bussadori

**Affiliations:** 1 Postgraduate Program in Rehabilitation Sciences, Universidade Nove de Julho (UNINOVE), R. Vergueiro, 235 – Liberdade, São Paulo/SP CEP 01504-001, Brazil; 2 Postgraduate Program in Health Systems Management, Universidade Nove de Julho (UNINOVE), R. Vergueiro, 235 – Liberdade, São Paulo/SP CEP 01504-001, Brazil

**Keywords:** Electromyography, Low-level laser therapy, Temporomandibular joint disorders, Temporomandibular joint

## Abstract

**Background:**

A number of problems involving the temporomandibular joint (TMJ) and associated structures can lead to temporomandibular disorder (TMD). The aim of the proposed study is to assess the effect of low-level laser therapy on occlusal contacts, mandibular movements, electromyography activity in the muscles of mastication and pain in adolescents with TMD.

**Methods/Design:**

A randomized, controlled, double-blind, clinical trial will be carried out involving 85 male and female adolescents between 15 and 18 years of age. The research diagnostic criteria for TMD will be used to assess all individuals who agree to participate. All participants will be submitted to a clinical examination and electromyographic analysis of the masseter muscles and anterior bundle of the temporal muscles bilaterally, to determine TMD. Based on the clinical findings, the participants will be classified as having or not having TMD. Those with TMD will be divided into four groups, three of which will receive low-level laser therapy and one of which will receive a placebo treatment. The treatments will involve the TMJ region alone, the masseter and temporal muscles alone, or both these regions together. The data will be submitted to descriptive statistical analysis. The chi-square test and Fisher’s exact test will be used to determine associations among the categorical variables. The Student’s *t* test and analysis of variance will be used for the comparison of mean electromyographic signals. Pearson’s correlation coefficients will be calculated for the analysis of correlations among the continuous variables.

**Trial registration:**

The protocol for this study has been submitted to Clinical Trials – registration number (NCT01846000).

## Background

Temporomandibular disorder (TMD) is a term used for a large number of clinical signs and symptoms that affect the muscles of mastication, the temporomandibular joint (TMJ) and associated structures [1-9]. Postural and occlusal abnormalities, parafunctional habits, and psychological factors can lead to TMD [2,4,5,8]. Orofacial pain is the most common complaint among individuals with this condition [2,4,5]. Other symptoms include sensitivity in the muscles of the head and neck region (including the muscles of mastication), pain in one or both TMJs, limited mandibular movements, joint noises, [1-7,10-14] headache, [1-6,10] dizziness, hearing loss and ringing in the ears [2-5]. The presence of symptoms can affect the quality of life and the social life of the patient.

The stomatognathic system includes the maxilla, mandible, teeth, TMJs, and muscles of mastication and is directly related to the cervical spine. The neuromuscular influences of the neck region and the function of mastication actively participate in mandibular movements and the positioning of the cervical spine. Balance and synchrony between the anterior and posterior muscles of the head and neck are needed to establish the resting position of the mandible [4,5].

Alterations in postural balance result in a change in electrical activity in the muscles of mastication, which can be determined using electromyography (EMG) [4]. Electromyographic analysis of these muscles can provide information regarding physiological abnormalities in the stomatognathic system of individuals with TMD in an effort to clarify the relationship between electrical activity and the mechanical response of the muscles [15,16].

Occlusal interferences are related to signs and symptoms of TMD [17,18], can affect chewing function and can lead to asymmetry in the stomatognathic system. Studies involving individuals with adequate occlusion (absence of dysfunction) have demonstrated that mastication occurs with symmetrical activity between the right and left masseter and temporal muscles. However, the muscle contraction pattern is altered and asymmetrical in individuals with occlusal interferences, as demonstrated by EMG during mastication [17].

Low-level laser therapy (LLLT) is a low-cost noninvasive treatment that has proven to be beneficial in the treatment of pain related to TMD [1-3,6,11,19-22]. The therapeutic effects of LLLT include the modulation of inflammatory processes, analgesia and cellular activity [1-3,6,7,11-13,19-25]. Despite the reported benefits [1-3,6,11,20-22], few studies have analyzed the effects of LLLT administered to adolescents with a diagnosis of TMD [1-3,6,7,11-13,20-25].

The aim of the proposed study is to assess the effect of LLLT on pain, occlusal contacts, mandibular movements, and electromyography activity in the masseter and temporal muscles in adolescents with TMD.

## Methods/Design

**Figure 1:**
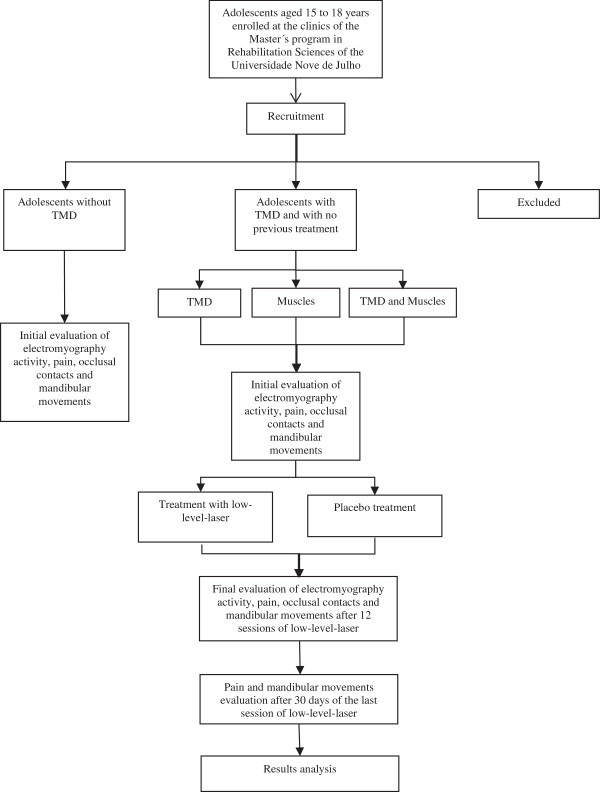
Flow chart of experimental design.

A randomized, controlled, double-blind, clinical trial will be carried out. The protocol for this study received approval from the Human Research Ethics Committee of the *Universidade Nove de Julho* (São Paulo, Brazil) under process number 40455. The legal guardians of the participants will sign a statement of informed consent authorizing participation in the study. Figure 1 summarizes the trial design.

### Subjects

Male and female adolescents enrolled at the clinics of the Master’s program in Rehabilitation Sciences of the *Universidade Nove de Julho* (Vergueiro Campus, São Paulo, Brazil) will be evaluated.

#### Inclusion criteria

Individuals aged 15 to 18 years with a diagnosis of TMD and a signed statement of informed consent will be included in the study.

#### Exclusion criteria

Individuals with dentofacial anomalies or incomplete permanent dentition to the second molar, or who are currently undergoing orthodontic or orthopedic treatment of the jaws, or who are undergoing psychological treatment or physical therapy and make use of a muscle relaxant, anti-inflammatory agent, or bite plate will be excluded from the study.

### Procedures

As the proposed study constitutes a randomized clinical trial, the recommendations of the Consolidated Standards of Reporting Trials (CONSORT) [26] will be followed to allow greater transparency and enhance the quality of the findings. The subjects will be allocated to five groups, as delineated in Table 1.

**Table 1:** Summary of experimental conditions

**Group**	**TMD**	**Laser application site**	**Therapeutic intervention**
I	Absent	—	Control group
II	Present	TMJ	LLLT
III	Present	Muscles of mastication	LLLT
IV	Present	TMJ and muscles of mastication	LLLT
V	Present	TMJ and muscles of mastication	Placebo

#### Diagnosis of temporomandibular disorder

Prior to any intervention, participants eligible for the study will be diagnosed with TMD, using the Research Diagnostic Criteria for Temporomandibular Disorders (RDC/TMD) questionnaire. Moreover, a specific clinical examination will be carried out by a single, previously trained, examiner. This examination will consist of palpation of the temporal, masseter, digastric and medial pterygoid muscles, palpation of the TMJs and an analysis of the mandible with the aid of a digital caliper to measure vertical and horizontal movements and a stethoscope to assess joint noises. The following will also be investigated: frequent headache, facial pain, fatigue, and difficulty during mastication, bruxism (clenching or grinding of teeth), psychological status, and parafunctional habits.

#### Surface electromyography

The EMG signal will be determined using a four-channel acquisition system (*EMG System do Brasil Ltda*) made up of a signal conditioning module, active bipolar electrodes with 20× pre-amplification, analog band-pass filter (20 to 500 Hz) and 120 dB common rejection mode. The sampling frequency will be 2 kHz. The signal will be digitized using an analog-to-digital converter with 16-bit resolution. The acquisition program will be EMGLab (*EMG System do Brasil Ltda*).

To capture the EMG of the right and left masseter and anterior temporal muscles, the skin will be cleaned with alcohol to diminish impedance and disposable, self-adhesive anterior surface electrodes (Ag/AgCl, Meditrace) measuring 10 mm in diameter will be attached to the belly of the muscle in the region with the greatest tone after the volunteer performs moderate intercuspation. The interelectrode distance will be 20 mm from center to center, as suggested by the European Recommendations for Surface Electromyography [27]. An electrode placed on the left wrist of the volunteer will be used as reference to impede the interference of external noise.

The EMG activity of the masseter and anterior temporal muscles will be read under three conditions: (i) at rest; (ii) during chewing (isotonic); and (iii) during maximum intercuspation (MI) (isometric). The signals referring to the resting and chewing conditions will be measured with no material placed between the upper and lower arches. During the readings, the volunteer will remain seated comfortably in a chair with hands resting on the thighs. Three readings will be made under each condition, with a three-minute rest interval between readings. The readings in the resting position will be taken first and each reading will last 15 seconds. The readings for the chewing condition will be taken next, with the volunteer simulating habitual chewing at a pace determined by a metronome adjusted to 60 beats a minute; each reading will last 15 seconds. The MI readings will then be performed. Three 10-second readings will be performed with no material placed between the arches, followed by three 5-second readings during maximum clenching effort (MCE) with a folded layer of Parafilm M® [28], 3 mm thick (15 mm × 35 mm) between the molars (bilaterally).

The EMG will be carried out before and after LLLT treatment.

### EMG signal processing

To determine EMG activity in the masseter and temporal muscles at rest and during MCE, the signal will be rectified by a mobile mean of 200 ms based on the root mean square (RMS) of the amplitude of the signal. For each condition, a 4-second range will be selected in which the RMS is the most constant. The mean RMS in this time interval (RMS_raw_) will be normalized by the RMS obtained during MCE (RMS_MCE_). The values referring to the resting and MI conditions will be expressed as a percentage of RMS_MCE_:

$$\%\mathrm{RMS}=\left({\mathrm{RMS}}_{\mathrm{raw}}/{\mathrm{RMS}}_{\mathrm{MCE}}\right)\times 100.$$

The EMG signal during chewing will be rectified and normalized using the peak of the signal followed by the calculation of the RMS. Signal processing will be performed using specific routines of the Matlab program, version 7.1 (MathWorks Inc., Natick, MA, USA).

#### Evaluation of occlusal contacts

The type of occlusion will be determined by the clinical examination based on the Angle classification [29], which is the most practical classification and is considered the gold standard in the literature. This system is based on anteroposterior relationships between the arches:

•Angle Class I (neutral occlusion): the mesiovestibular cusp of the permanent upper first molar articulates with the buccal sulcus of the permanent lower first molar.

•Angle Class II (distal occlusion): the mesial sulcus of the permanent lower first molar articulates posteriorly to the mesiobuccal cusp of the permanent upper first molar.

•Angle Class III (mesial occlusion): the mesial sulcus of the permanent lower first molar articulates anteriorly to the mesiobuccal cusp of the permanent upper first molar.

The T-Scan® III system will be used to record occlusal contact points. This system is composed of contact sensors connected to a USB port of a computer. Occlusal forces are recorded using a specific software program. The volunteer will be positioned in a chair at 90 degrees such that the Camper plane is parallel to the ground and will be instructed to perform MI. The T-Scan® III allows the simultaneous recording and imaging of the distribution of occlusal forces and time sequences, as well as premature contacts and interferences in the occlusion dynamics [30].

Determination of occlusal contact points will be made at the beginning and end of treatment as well as 30 days after the last session.

#### Evaluation of mandibular range of motion

The guidelines of the International Association for Dental Research will be followed when evaluating the range of motion of the mandible; the guidelines recommend a single measurement of each movement [31]. The volunteer will be instructed to open the mouth as wide as possible. Maximum voluntary mouth opening (distance between upper and lower central incisors) will be recorded with the aid of a digital caliper. The volunteer will then be instructed to exert pressure on the lower teeth with the index and middle finger to obtain maximum passive mouth opening and move the mandible to the right and left for the determination of excursion (distance between upper and lower midpoints). These procedures will be carried out at the beginning and end of treatment as well as 30 days after the last session [1,2].

#### Evaluation of pain

A visual analog scale will be used before and after LLLT as well as 30 days after the last session to record pain upon palpation of the masseter and temporal muscles [1,2,9,24]. This is a numeric rating scale that runs from 0 (absence of pain) to 10 (worst pain imaginable) [1].

#### Application of low-level laser therapy

A gallium-aluminum-arsenide laser (Twin Flex Evolution®, MM Optics) will be employed for the LLLT and placebo treatment. The sessions will be held in a reserved, noise-free room. The volunteer will be positioned with the Frankfurt plane parallel to the ground. The active tip of the laser will be covered in disposable plastic wrap to avoid cross-contamination and for hygiene reasons. The operator will wear suitable protective clothing. The skin at the site to be irradiated will have previously been cleaned with 70% alcohol. Both the operator and volunteer will use protective eyewear.

Two sessions of LLLT or placebo treatment will be carried out over six weeks (a total of 12 sessions). The device will be calibrated with a wavelength of 780 nm, an energy density of 25 J/cm^2^, a power of 50 mW, and a power density of 1.25 W/cm^2^. The exposure time will be 20 s per point, resulting in a total energy of 1 J per point. The spot application method will be used: the laser will have a conventional tip in contact with the skin and will cover an area of 0.04 cm^2^, as suggested by Venezian *et al*. [24] and Carvalho *et al*. [14]. Three of the four groups with TMD will receive LLLT and one will receive a placebo treatment:

1. TMJ region: five points around the TMJ [7,13], totaling 5 J.

2. Masseter and temporal muscles: three points on the masseter (upper, middle and lower) and one point on the anterior temporal [24], totaling 4 J.

3. Mixed application: TMJ and muscles of mastication [12,14], resulting in 9 J.

4. Placebo treatment: the same equipment will be used with a pen that emits a red guide light and a warning sound, but without the emission of a laser beam.

### Sample size calculation

Based on data from the literature [24], using the EMG signal of the masseter muscles, and following LLLT as the basis, with mean values of 13.4 (standard deviation: 2.8) and 11.9 (standard deviation: 1.9) individuals per group, for the experimental study and comparison of means with equal samples (hypothesis test), considering a 5% level of significance (*P* < 0.05) and 80% test power, the sample size was determined to be 15 individuals per group, using the DINAM 1.0 program [32].

Seventeen individuals will be selected for each group to compensate for possible losses.

#### Organization and statistical treatment of data

The data will be tabulated and treated using the SPSS 12.0 program for Windows and will be submitted to descriptive statistical analysis. The chi-square test and Fisher’s exact test will be used to determine associations among the categorical variables. The Student’s *t* test for paired data will be used to analyze the averages of electromyographic and occlusal contacts before and after treatment. The Student’s *t* test and analysis of variance will be used to compare mean electromyographic signals and occlusal contacts between different groups. For data that are not normally distributed, the differences before and after treatment in the same group and between groups will also be analyzed, using the Mann–Whitney and Wilcoxon paired tests. Pearson’s correlation coefficients will be calculated for the analysis of correlations among the continuous variables. The level of significance will be set to 5% (*P* < 0.05).

## Discussion

Signs and symptoms of TMD are found in a large portion of the population. The pain that often accompanies this condition can compromise mandibular movements and lead to a reduction in quality of life. Bad posture, malocclusion, parafunctional habits, and other clinical conditions can lead to the development of TMD, which may also be associated with psychological factors [2,4,5,8].

Orofacial pain is the most common complaint of individuals with TMD. This pain can vary from mild to extreme discomfort and may result from the increase in muscle activity [2,4,5]. Surface EMG assists in the diagnosis of TMD by allowing the assessment of muscle function and dysfunction in the resting position as well as during occlusion and mastication. Indeed, *in vivo* analysis of muscle physiology is important to the diagnosis of TMD and the monitoring of this condition throughout the therapeutic process [29].

Owing to the complexity and multifactor etiology of this TMD, a number of treatment options have been proposed, such as LLLT, acupuncture, ultrasound, massage therapy, psychological treatment, drug therapy, and the use of a bite plate [1,9,24,25]. LLLT is a low-cost, noninvasive form of treatment that offers pain relief, a reduction in inflammation and enhanced tissue regeneration [1-3,6,11,20-22]. While a number of studies report the use of LLLT for the treatment of TMD, [1-3,6,7,11-13,20-25] few papers have addressed treatment in adolescents. Thus, the investigation of the effects of LLLT in the adolescent population with TMD is of extreme importance to outlining an effective treatment protocol for pain relief and improving the quality of life of affected individuals.

## Trial status

The proposed study is currently in the recruitment phase, and individuals are being diagnosed with TMD using the RDC-TMD questionnaire.

## Abbreviations

LLLT: Low-level laser therapy; MCE: Maximum clenching effort; MI: Maximum intercuspation; RMS: Root mean square; TMD: Temporomandibular disorder; TMJ: Temporomandibular joint.

## Competing interests

The authors declare that they have no competing interests.

## Authors’ contributions

CHLG and SKB conceived and designed the study and contributed to drafting the manuscript. DABG, LJM, and FP contributed to the design of the study. PFCS and DSA contributed to writing the manuscript. RAMF, KPSF, and RA revised the manuscript. All authors read and approved the final manuscript.
